# Technical Note: A novel dosimeter improves total skin electron therapy surface dosimetry workflow

**DOI:** 10.1002/acm2.12880

**Published:** 2020-04-19

**Authors:** Irwin I. Tendler, Petr Bruza, Michael Jermyn, Jennifer Soter, Gregory Sharp, Benjamin Williams, Lesley A. Jarvis, Brian Pogue, David J. Gladstone

**Affiliations:** ^1^ Thayer School of Engineering Dartmouth College Hanover NH USA; ^2^ DoseOptics LLC Lebanon NH USA; ^3^ Department of Radiation Oncology Massachusetts General Hospital Boston MA USA; ^4^ Department of Medicine Geisel School of Medicine Dartmouth College Hanover NH USA; ^5^ Norris Cotton Cancer Center Dartmouth‐Hitchcock Medical Center Lebanon NH USA

**Keywords:** dosimeter, efficiency, optical imaging, OSLD, scintillator, surface dosimetry, workflow

## Abstract

**Purpose:**

The novel scintillator‐based system described in this study is capable of accurately and remotely measuring surface dose during Total Skin Electron Therapy (TSET); this dosimeter does not require post‐exposure processing or annealing and has been shown to be re‐usable, resistant to radiation damage, have minimal impact on surface dose, and reduce chances of operator error compared to existing technologies e.g. optically stimulated luminescence detector (OSLD). The purpose of this study was to quantitatively analyze the workflow required to measure surface dose using this new scintillator dosimeter and compare it to that of standard OSLDs.

**Methods:**

Disc‐shaped scintillators were attached to a flat‐faced phantom and a patient undergoing TSET. Light emission from these plastic discs was captured using a time‐gated, intensified, camera during irradiation and converted to dose using an external calibration factor. Time required to complete each step (daily QA, dosimeter preparation, attachment, removal, registration, and readout) of the scintillator and OSLD surface dosimetry workflows was tracked.

**Results:**

In phantoms, scintillators and OSLDs surface doses agreed within 3% for all data points. During patient imaging it was found that surface dose measured by OSLD and scintillator agreed within 5% and 3% for 35/35 and 32/35 dosimetry sites, respectively. The end‐to‐end time required to measure surface dose during phantom experiments for a single dosimeter was 78 and 202 sec for scintillator and OSL dosimeters, respectively. During patient treatment, surface dose was assessed at 7 different body locations by scintillator and OSL dosimeters in 386 and 754 sec, respectively.

**Conclusion:**

Scintillators have been shown to report dose nearly twice as fast as OSLDs with substantially less manual work and reduced chances of human error. Scintillator dose measurements are automatically saved to an electronic patient file and images contain a permanent record of the dose delivered during treatment.

## 1 INTRODUCTION

Currently, surface dosimetry clinical workflow during Total Skin Electron Therapy (TSET) presents numerous opportunities for human error. Steps such as manual input of dosimeter anatomical location as well as tracking of unique dosimeter identifications during transferring and handling can lead to dosimeter mismatch errors.^1^ When coupled with time‐intensive tasks such as serial readout, registration, and in some cases annealing, this results in a cumbersome process for obtaining surface dose measurements during TSET. The time burden of this process can sometimes lead clinical the team to conduct fewer surface dose measurements, or even in the extreme, discourage treatment centers from adopting TSET – an effective, but lengthy, method for treating cutaneous lymphoma. For example, a recent study from a high patient volume center has shown that clinicians have reduced the total number of dosimetry sites in order to save time in (thermo‐luminescent detectors) dosimeter preparation and readout.^2^ We suggest that instead of minimizing the number of measured dosimetry sites, one can reduce the amount of time required to conduct surface dosimetry measurements via optimization of the workflow.

Previous studies have shown that capturing light emission from plastic discs attached to the skin surface during radiation therapy is an accurate, non‐invasive, remote, and efficient method for measuring surface dose.^3^, ^4^, ^5^, ^6^, ^7^ Images are recorded using a time‐gated and intensified CMOS camera synchronized to linac pulses. A custom image processing algorithm converts pixel intensities to surface dose using a fitting function and external calibration factor. Scintillator dosimeters are resistant to radiation damage (tested up to 20 kGy), have a maximum wavelength of emission at 422, induce a build‐up effect comparable to Optically Stimulated Luminescence Detectors (OSLD, 4.9%), and, within the context of TSET, do not require a correction factor for temperature, dose rate, camera‐dosimeter distance, or camera‐dosimeter angle.^4^

**Fig. 1:**
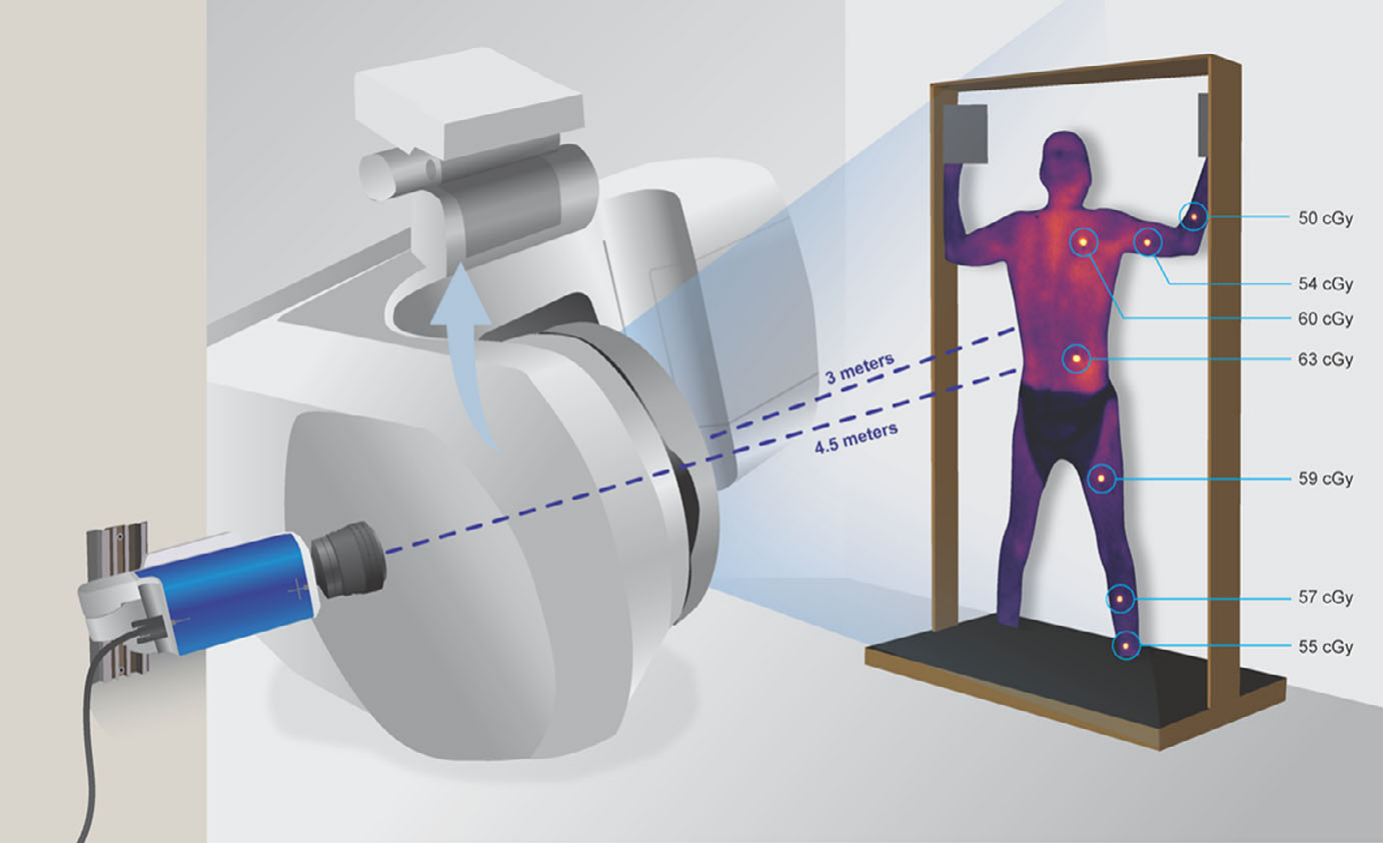
Schematic of the imaging and patient treatment (TSET) setup. Camera‐patient and linac‐patient (SSD) distances are also provided. Doses measured by scintillators at the 7 measured dosimetry sites are provided for a sample posterior‐anterior irradiation (300 MU for this single Stanford TSET position, image is a cumulative sum of all frames). To note, each Stanford TSET position required administration of two separate irradiations, one with the gantry at 289.5° and 250.5°; these two angles were optimized to achieve best vertical dose uniformity and need to be determined from on‐site measurements. For illustrative purposes, both fields are shown as a sum with an arrow indicating the trajectory of gantry movement. For phantom imaging, the patient treatment stand was removed and the phantom was placed on a stand 3 meters (SSD) away from the linac at the center of the beam while the gantry was held at 90^o^.

By virtue of being positioned perpendicular to the patient, the camera offers a unique perspective view of the treatment field, allowing for real‐time simultaneous monitoring of patient positioning with surface dose, and including compliance (maintaining the Stanford Technique positions, keeping hands open, etc.) during treatment otherwise impossible from standard CCTV viewing angles. Figure 1 shows how the camera is positioned with respect to the linac and patient. This scintillator imaging system also inherently records the location of each dosimeter during acquisition, so that the delivered versus planned treatment dose can be estimated without any ambiguity about the anatomical location of dosimeter placement.^8^, ^9^ In this study, the functional workflow of scintillator imaging is quantitatively examined and compared to that of a standard method, OSLDs.

## 2 METHODS

Both the phantom and patient were irradiated with a 6 MeV High Dose Total Skin Electron beam using a Varian Trilogy linear accelerator (linac, Varian Medical Systems, Palo Alto, CA).

### 2.A Camera setup and dosimeters

All imaging was conducted using a time‐gated and intensified C‐Dose Research (DoseOptics LLC, Lebanon, New Hampshire) camera coupled to 50 mm Nikkon 50‐mm f/2.8 AF lens (Nikon Inc., Tokyo, Japan) lens. Compared to previous work, differentiating features of the imaging setup included use of remote trigger unit (eliminating the need for the “trigger cable” mentioned in previous publications) and custom wall‐mount for the camera, see Figure 1.^4^, ^10^, ^11^ The camera was mounted facing the TSET treatment stand at a height of 1.2 m behind the gantry head; with the gantry positioned at 270°, the camera mount was 1.3 m laterally to the right side (measured from the edge of the gantry head). The center of the field of view was aimed at a height of 1.3 m from the ground representing (approximately) patient mid‐line. The design and specifications of the scintillators has been previously discussed in detail.^3^, ^5^, ^6^, ^7^, ^12^ Scintillator discs (15 mm Ø × 1 mm thick) were custom machined out of EJ‐212 plastic (Eljen Technologies, Sweetwater, TX) and coated along the rear face and edge with EJ‐510 reflective paint (Eljen Technologies, Sweetwater, TX) to minimize the impact of Cherenkov light generated from tissue underneath the discs. Given the thickness of the scintillators and considering the energy of the TSET beam (6 MeV), total light output from the disc provides an effective point of measurement very close to the skin surface, similar to OSLDs.^13^ During imaging, the camera was located 4.5 m from the patient and phantom; sconce lighting (light intensity remained unchanged compared to standard clinical operations) was used to illuminate the room during treatment and imaging. Online image processing steps and the methodology for generating the required scintillator calibration factor (TSET‐specific) have been previously reported.^4^, ^5^, ^6^ Following image acquisition, a custom MATLAB (Mathworks, Natick, MA) algorithm was used to convert pixel intensities to dose using a Precision 5530 laptop (Dell Inc., Round Rock, TX) running a i9‐8950HK processor (Intel, Santa Clara, CA) with 32 GB of RAM and a 970 PRO SSD (Samsung, Seoul, South Korea).^4^

### 2.B Workflow assessment

An Android 9.0 embedded timer (Google LLC, Mountain View, CA) was used for making time measurements during workflow assessment. The following steps were timed for surface dosimetry‐associated workflow: daily QA, dosimeter preparation, dosimeter attachment, dosimeter removal, dosimeter registration, and data readout.

### 2.C Phantom imaging

Directly adjacent pairs of scintillators and OSLDs (nanoDot, Landauer Inc, Glenwood, IL) were attached to a flat‐faced phantom (n = 4) and irradiated at a 3 m source‐surface‐distance (SSD) one at a time. Surface dose measurements obtained by scintillators were compared to those of OSLDs.

### 2.D Patient imaging

All human imaging was completed on an Institutional Review Board (IRB) approved protocol; informed patient consent was obtained and all procedures followed this protocol. Surface dose at 7 anatomical locations (upper arm, lower arm, chest, midsection, mid‐thigh, mid‐shin, and upper foot) was tracked over 5 treatment days (2 poster‐anterior and 3 anterior‐posterior position) of a patient undergoing TSET. Scintillator discs (with a protective coating and rear‐sided adhesive patch) were attached at each of these sites and an OSLD was placed directly adjacent to each. Previously, a detailed description of surface dose measured by scintillator versus OSLD for a number of different anatomical locations has been described.^5^

## 3 RESULTS & DISCUSSION

### 3.A Dosimetry results

As reported in previous studies, surface doses measured by scintillator and OSLDs were compared for both phantom and patient data sets.^3^, ^5^, ^6^, ^7^ In phantoms, scintillators and OSLDs surface doses agreed within 3% for all data points. During patient imaging it was found that surface dose measured by OSLD and scintillator agreed within 5% and 3% for 35/35 and 32/35 dosimetry sites, respectively.

### 3.B Summary table

Data for phantom studies was collected on a per dosimeter basis and is shown in Table 1 as an average of these measurements for each step of the workflow process. Timing individual steps during patient imaging was not possible, and as such, times were measured for a group (n = 7) of dosimeters. Thus, patient data in Table 1 is an average of time spent using a group of dosimeters throughout the surface dosimetry workflow. Each entry in Table 1 also reports the standard deviation associated with each set of average measurements.

**Table 1:** Timed steps of the surface dosimetry workflow, all numbers shown as mean ± SD in seconds. Phantom data is reported per dosimeter while patient data is for a group of n = 7 dosimeters.

Scintillator (sec)			OSLD(sec)	
Phantom	patient	Workflow	Phantom	patient
0	0	Daily QA	120 ± 20	120 ± 20
5 ± 2	39 ± 7	Dosimeter preparation	11 ± 3	83 ± 5
3 ± 2	29 ± 5	Attachment	2 ± 1	35 ± 4
2 ± 1	11 ± 2	Removal	2 ± 1	11 ± 2
0	0	Registration	17 ± 10	125 ± 13
68 ± 4	307 ± 9	Readout	50 ± 13	380 ± 25
78	386	Total (sec)	202	754

### 3.C Daily QA

The microSTARii OSLD reader (nanoDot, Landauer Inc, Glenwood, IL) requires a standard daily QA procedure which involves reader stability and constancy testing.^14^ Overall, it was found that taking 10 readings with no OSLD present followed by scanning and reading a constancy OSLD 10 times took, on average, a total of 120 ± 20 sec. The scintillator dosimetry system does not have an explicit daily QA process; however, reference discs attached directly to the patient treatment stand inherently provide a measurement of camera stability and reproducibility – this information is available in each acquired image. Though, if so desired, implementation of a routine QA measurement would not be difficult to accomplish – scintillator light output can be tracked a given location in the radiation field and compared to readings from a standard dosimetry device (OSLD, diode, etc.) over time.

### 3.D Dosimeter preparation, attachment, and removal

Commercially available OSLDs are sold in see‐through plastic sachets (containing a light‐tight plastic holder encapsulating the OSLD active element), furthermore, distinguishing one dosimeter from another by eye is difficult (small‐sized identifier is written along the dosimeter edge).^15^ Thus, a marker was used to label each dosimeter and two pieces of medical tape were applied. Preparation of OSLDs was found to take on average 11 ± 3 sec per dosimeter, an average time of 83 ± 5 sec was measured during preparation for patient surface dosimetry. Scintillators have a protective coating allowing for application of a double‐sided adhesive backing directly to their rear face without impacting light out; detailed description showing cross section of the scintillator have been previously described.^6^ Scintillators do not need to be marked prior to usage as the location of the dosimeter is inherently recorded in each image. Application of the adhesive backing took on average 5 ± 2 sec per disc and an average time of 39 ± 7 sec was recorded for patient surface dosimetry preparation.

Both scintillators and OSLDs were placed on the phantom surface and patient’s skin by hand in the desired locations. For scintillators, the adhesive backing was peeled off prior to attachment and average time for phantom and skin application was 3 ± 2 and 29 ± 5 sec, respectively. Average time for OSLD application was on average 35 ± 4 and 2 ± 1 sec for patient and phantom testing, respectively. Both dosimeters were removed from the phantom and patient surfaces by hand simultaneously resulting in an average removal time of 2 ± 1 sec for individual dosimeters and 11 ± 2 sec for the sets of dosimeters during patient imaging.

### 3.E Imaging workflow

To acquire images, one simply needs to plug the power supply of the camera into the wall, remove the lens cap prior to imaging, enter the desired file name into commercial software (DoseOptics, Lebanon, NH), and click start. To note, the camera does not have a warm‐up period unlike the OSLD reader which requires 1 h of on‐time prior to usage; warm‐up can be avoided if the OSLD reader is left continuously on.^16^ Since the camera triggers directly off of linac pulses, one can simply leave the camera in “standby” as it will trigger once the radiation beam is activated automatically – triggering and data collection are conducted wirelessly. Previously, manual positioning of a tripod was required prior to imaging, this aspect of the workflow has been eliminated by use of a wall‐mounted camera setup.^5^

### 3.F Dosimeter registration and readout

OSLDs must be registered with the microSTARii database prior to readout; this creates a unique record of the dosimeter and assigns it a background count value. By design, this is done one dosimeter at a time by scanning the barcode on each OSLD using a QR reader.^17^ Average time to register a single OSLD was 17 ± 10 sec and 125 ± 13 sec for a group of 7. When considering a batch of scintillators, one does not need to keep track of individual scintillators as dosimeter‐dosimeter variation has been shown to be 0.3%  ± 0.2% and they have been tested to be unaffected by radiation damage to 15 kGy.^4^ Furthermore, when also accounting for camera stability (2% ± 1%), the uncertainty budget of TSET scintillator dosimetry system is comparable to that of a clinically commissioned nanoDot OSLDs (1.6% – 4.9% depending on calibration conditions and operator experience).^1^, ^5^ The publicly‐available algorithm for converting pixel intensities to dose was modified slightly – the user no longer needs to manually enter coordinates of disc centroid. Once the algorithm is run, a cumulative image of the patient data set is generated, and the user simply clicks on the discs in the image they want to include in analysis.

The average time to read out a single scintillator dosimeter during phantom testing was on average 68 ± 4 sec while patient surface dosimetry readout (n = 7 dosimeters) took on average 307 ± 9 sec. For OSLDs, readout (standard 4x readings) for a single dosimeter took 50 ± 13 sec and 380 ± 25 sec for patient surface dosimetry readings.

A key advantage to using scintillators is that the time to readout data does not linearly scale with the number of dosimeters used. Furthermore, the time burden of individually reading out each dosimeter is also minimized – one can simply let the algorithm compute surface dose measurements without requiring continual manual input, as is the case for OSLDs. The readout time of the OSLDs requires consistent manual input (each OSLD must be individually scanned, loaded, readout, and a “note” must be entered) and specialized equipment (microSTARii reader). Scintillator images can be analyzed on desktop or laptop (as they were in this study), there is also no designated time window for processing whereas OSLD have a decay factor after irradiation**.** ^18^ Use of a faster processor, SSD, and additional RAM would speed up the scintillator readout process.

Reusability of OSLDs is not recommended by the manufacturer. However, in an effort to minimize cost and material waste, research groups have created a multistep process which involves manipulation of the dosimeter, exposure to a high‐intensity light source, and tracking of lifetime exposure (maximum exposure of 10 Gy).^19^, ^20^, ^21^ Scintillators are coated with a protective coating and can be sanitized using standard clinical procedures; coupled with their resistance to radiation damage allows for dosimeter reuse.

### 3.G Cost analysis

The current cost of the CDose Research imaging system and affiliated software is $44,800, this figure is comparable to the cost of a microSTARii OSLD reader ($29,000, Landauer Inc Glenwood, IL). The manufacturing cost for each scintillator disc is currently $20 – $30 for batches of 10 – 20 dosimeters, respectively. However, if scintillator production was to be scaled, the cost per disc is expected to decrease and be similar to that of nanoDot OSLDs (currently priced at approximately $12 per dosimeter).

## 4 CONCLUDING REMARKS

One of the main limitations of the scintillator dosimetry system is that in order to measure surface dose at a given anatomical location, the dosimeter must be in the field of view of the camera. However, as a potential solution to this problem, a multi‐camera method that could enable a 360° view of the patient is currently under development. Additionally, by incorporating mirrors into this imaging setup, surface dose in locations out of the line of sight of the current system could potentially be measured using scintillators e.g. areas receiving scatter radiation outside of the primary beam and field of view of the camera. The scintillators utilized in this study can be sanitized and reused patient‐to‐patient, however, long term use may lead to lead to wear‐and‐tear issues such as deep scratches and chipping of the protective coating. Nevertheless, the scintillators used in this report have been heavily used for phantom and patient imaging and over 8 months and no noticeable issues have arisen. Overall, the results of this study show that scintillator dosimeters can measure surface dose during TSET nearly twice as fast standard OSLDs without sacrificing accuracy.

## FUNDING INFORMATION

This work was sponsored by NIH grants R44 CA199836, R01 EB023909, and P30 CA023106.

## CONFLICT OF INTEREST

M. Jermyn is an employee and B. Pogue is president of DoseOptics LLC. P Bruza is principal investigator in SBIR subaward B02463 (prime award NCI R44CA199681, DoseOptics LLC).
